# Measurement of the Three-Dimensional Shape of Discontinuous Specular Objects Using Infrared Phase-Measuring Deflectometry

**DOI:** 10.3390/s19214621

**Published:** 2019-10-24

**Authors:** Caixia Chang, Zonghua Zhang, Nan Gao, Zhaozong Meng

**Affiliations:** 1 State Key Laboratory of Reliability and Intelligence of Electrical Equipment, Hebei University of Technology, Tianjin 300130, China; ccaixia@foxmail.com; 2 School of Mechanical Engineering, Hebei University of Technology, Tianjin 300130, China; ngao@hebut.edu.cn (N.G.); zhaozong.meng@hebut.edu.cn (Z.M.)

**Keywords:** infrared phase-measuring deflectometry (IR-PMD), three-dimensional (3D) shape measurement, specular objects, fringe reflection, fringe projection, absolute phase

## Abstract

Phase-measuring deflectometry (PMD)-based methods have been widely used in the measurement of the three-dimensional (3D) shape of specular objects, and the existing PMD methods utilize visible light. However, specular surfaces are sensitive to ambient light. As a result, the reconstructed 3D shape is affected by the external environment in actual measurements. To overcome this problem, an infrared PMD (IR-PMD) method is proposed to measure specular objects by directly establishing the relationship between absolute phase and depth data for the first time. Moreover, the proposed method can measure discontinuous surfaces. In addition, a new geometric calibration method is proposed by combining fringe projection and fringe reflection. The proposed IR-PMD method uses a projector to project IR sinusoidal fringe patterns onto a ground glass, which can be regarded as an IR digital screen. The IR fringe patterns are reflected by the measured specular surfaces, and the deformed fringe patterns are captured by an IR camera. A multiple-step phase-shifting algorithm and the optimum three-fringe number selection method are applied to the deformed fringe patterns to obtain wrapped and unwrapped phase data, respectively. Then, 3D shape data can be directly calculated by the unwrapped phase data on the screen located in two positions. The results here presented validate the effectiveness and accuracy of the proposed method. It can be used to measure specular components in the application fields of advanced manufacturing, automobile industry, and aerospace industry.

## 1. Introduction 

With the advent of the information age, optical methods for the acquisition of three-dimensional (3D)-shape information of objects has an increasingly important role in various application fields, such as advanced manufacturing, automotive industry, aerospace industry, and so on. Due to the advantages of high accuracy, full-field, noncontact acquisition, and quick data processing, optical 3D shape acquisition has been one of the major measurement techniques, especially suitable for the detection of high-quality and vulnerable surfaces [1,2,3,4,5,6]. The existing optical 3D shape measurement techniques are mainly applied to measure diffuse object surfaces [7,8,9,10]. However, many object surfaces exhibit special properties, such as having reflective identity and being specular in aerospace and automotive applications. Therefore, the investigation of 3D measurement methods to obtain shape information of specular surfaces has attracted extensive research efforts. 

Phase-measuring deflectometry (PMD)-based methods have been widely used in 3D shape measurement of specular objects, because they offer the advantages of a large dynamic range, high accuracy, and full-field, noncontact, and automatic data processing [11,12,13]. The fundamental principle of the PMD method is the fringe reflection technique. The existing PMD methods can be broadly categorized into two kinds: classical PMD and improved PMD. The classical PMD calculates the gradient distribution of specular surfaces by displaying sinusoidal fringe patterns, and then the 3D shape can be obtained by integrating the gradient data. Firstly, the sinusoidal fringe patterns are displayed on a digital screen, such as a liquid-crystal display (LCD). These fringe patterns are reflected by the specular object and are deformed with respect to the gradient and height variation of the measured surface. Secondly, the deformed fringe patterns are captured by an imaging device, such as a charge-coupled device (CCD) camera. The phase data are calculated from the captured fringe patterns. Thirdly, the gradient data are obtained after calibrating the measuring system to build the relationship between phase and gradient. Finally, by integrating the gradient data, the 3D shape information of the specular surface is reconstructed [14]. Therefore, the classical PMD methods cause cumulative errors during gradient data integration. More importantly, they cannot be used to measure multiple discontinuous and/or isolated specular surfaces. Many improved PMD methods have been studied to overcome these challenging problems in classical PMD methods. Zhao et al. [15] proposed a PMD-based method by using two reference screens for measuring a spherical mirror. The mirror surface was reconstructed by numerical integration. Guo et al. [16] proposed an improved PMD to determine the corresponding reflected rays by calibrations without knowing the incident rays. This method provides a new tool for quantitative measurements of aspheric surfaces in full field of optical manufacturing. Xiao et al. [17] presented a PMD calibration method using a markerless flat mirror to simplify the system calibration and reduce the error of the system calibration and the image noise. Huang et al. [18,19,20] put forward a method called modal PMD (MPMD) by using zonal integration to reconstruct the height residual for compensation. In order to minimize the discrepancy between the reprojection in ray tracing and the actual measurement, the surface height and gradient were represented by mathematical models and updated by optimizing the model coefficients. A direct PMD (DPMD) method was proposed to directly build the relationship between absolute phase maps and depth data by a mathematical model [21,22,23]. The DPMD method eliminated the procedure of gradient integral and therefore reduced the cumulative error. 

The existing PMD methods utilize visible light, but the measurement of specular surfaces is sensitive to ambient light. Therefore, the reconstructed 3D shape data are affected in the actual measurement. Moreover, visible light cannot create enough specular reflection to apply deflectometry on many visually rough metal surfaces. To measure diffusely specular surfaces, Höfer et al. [24] presented a method using infrared (IR) deflectometry. The technique enables the application of established methods and algorithms from conventional deflectometry, broadening the range of surfaces and materials that can be tested and has a wide range of applications in many fields. H. Toniuc et al. [25] presented an IR deflectometry method involving the use of a bi-directional grid to generate IR grid patterns; black squares were directly printed onto 1.5 mm-thick brushed aluminum plates. This method yields the classical advantages of deflectometry and allows the direct measurement of slopes. However, because the two methods do not use fringe patterns to calculate phase information, they are not PMD methods. Therefore, the measured accuracy when using the existing IR deflectometry is not high. Moreover, the two methods cannot be used to measure discontinuous specular objects. 

To reduce the effects of ambient light and measure discontinuous specular objects, a novel infrared PMD (IR-PMD) method is proposed that established a direct relationship between absolute phase and depth data. The invention patent filed of this method has been authorized [26]. In addition, a new calibration method of the measurement system is proposed by combining fringe projection and fringe reflection. The remainder of this paper is organized as follows. Section 2 describes the principle of measurement and calibration of the proposed IR-PMD method. In Section 3, experiments, results of the 3D shape reconstruction, and performance analysis are presented in details. Conclusions are drawn in Section 4.

## 2. Principle

Because there is no available IR display device, a projector was used to project invisible sinusoidal fringe patterns onto a ground glass to be regarded as an IR digital screen. In order to measure discontinuous specular objects, a translating stage moved the IR digital screen to two different positions along the normal direction of the screen surface. IR fringe patterns were reflected by the measured specular surfaces, and the deformed fringe patterns were captured by an IR camera. The multiple-step phase-shifting algorithm and the optimum three-fringe number selection method [27,28] were applied to the deformed fringe patterns to obtain wrapped and unwrapped phase data pixel by pixel, respectively. A plane mirror was used for geometric calibration. Part of the mirror surface was sprayed for the purpose of using the fringe projection technique to calibrate the system parameters. After calibration, 3D shape data of specular objects having discontinuous surfaces could be directly calculated from the unwrapped phase data in two screen positions. The following subsections elaborate the details of the proposed measurement method and the geometric calibration.

### 2.1. Measurement Method

**Figure 1:**
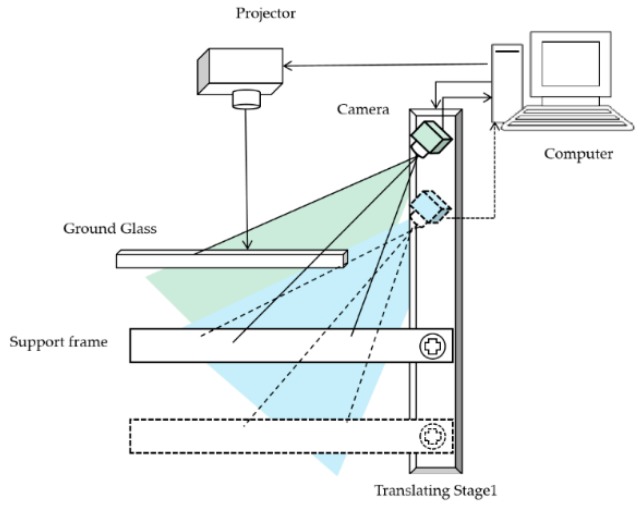
Measurement principle of the proposed infrared phase-measuring deflectometry (IR-PMD) method.

In order to directly obtain the 3D shape of specular objects from the deformed fringe patterns, a measurement method was developed to display IR sinusoidal fringe patterns onto an IR digital screen located in two different positions, as illustrated in Figure 1. The developed method uses a projector, an IR camera, a ground glass, a translating stage (called stage1), and a support frame. The support frame is used to place the measured specular objects. This method replaces the IR digital screen with the projector projecting IR fringe patterns onto the ground glass, and replaces two IR digital screens by moving the IR camera together with the measured specular objects to two different positions. As shown in Figure 1, the solid line is the position of the IR camera and the support frame before moving, and the dotted line is their position after moving. Because of the good diffusivity to light and high light transmittance of the ground glass, the IR camera can clearly capture the IR fringe patterns both on the ground glass and on the measured specular objects.

**Figure 2:**
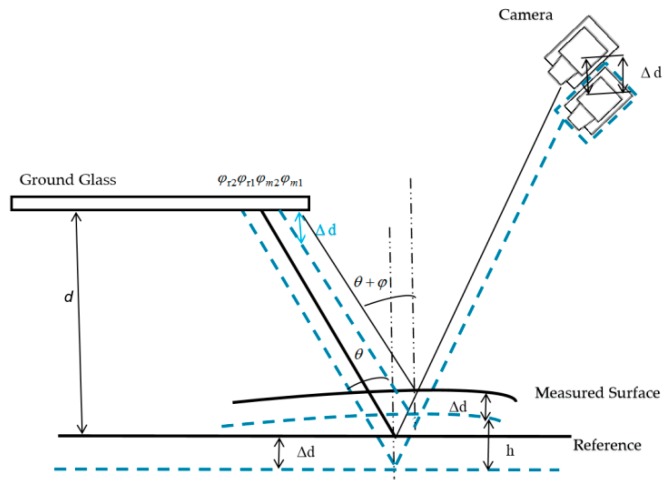
Schematic diagram of the IR-PMD method.

Figure 2 illustrates the schematic diagram of the IR-PMD method. A plane perpendicular to the projecting axis of the projector was chosen as a reference. The parameter *d* is the distance between the ground glass and the reference plane, *h* is the height of the measured specular surface with respect to the reference, ∆*d* stands for the moved distance by the translating stage1 during the measurement procedure. The absolute phases *φ*_r1_ and *φ*_m1_ of the two incident rays are denoted on the ground glass, *θ* is the angle between the incident ray and the normal vector of the reference plane, *θ* + *φ* is the angle between the incident ray and the normal vector of the measured specular surface. The IR camera with the measured specular surface was moved to another position by using the translating stage1. The positional relationship diagram after moving a distance ∆*d* is illustrated by the dotted line in Figure 2. Two incident rays of light are displayed and reflected into the IR camera by the measured surface. The two incident rays correspond to the same reflection light. The absolute phases *φ*_r2_ and *φ*_m2_ of the two incident rays are denoted on the ground glass.

According to the triangle similarity relationship, the following geometric relations can be obtained

(1) q(φm1−φm2)2π=Δdtan(θ+φ)

(2) q(φr1−φr2)2π=Δdtanθ

(3) Δl=φr1−φm12π

(4) (d+h)tanθ=(d−h)tan(θ+φ)−Δl

From the above Equations (1)–(4), the depth h of the measured surface is

(5) $$h=\frac{\Delta d\left(\varphi_{r1}-\varphi_{m1}\right)+d\left[\left(\varphi_{r1}-\varphi_{r2}\right)-\left(\varphi_{m1}-\varphi_{m2}\right)\right]}{\left(\varphi_{r1}-\varphi_{r2}\right)+\left(\varphi_{m1}-\varphi_{m2}\right)}$$

Equation (5) shows that the depth can be directly calculated from the captured fringe patterns only if the parameters ∆*d*, *d*, and absolute phase information on the reference plane are determined beforehand. Equation (5) is a relational expression containing only absolute phase data and depth information. The factors with gradient information in the equations are eliminated in the simultaneous process, thus the influence of the angle and the error accumulation in the gradient integral process is reduced. Therefore, the proposed method can be used to measure specular objects having isolated and/or discontinuous surfaces without an integration procedure.

### 2.2. Geometric Calibration

In order to obtain the accurate 3D shape of specular surfaces, an important step is to build the relationship between the absolute phase map and the depth data, which is known as geometric calibration. As shown in Equation (5), the geometric calibration of the IR-PMD method needs to determine two distances *d* and ∆*d*. In the proposed method, ∆*d* is a known quantity representing the distance moved by the translating stage1 during the procedure of measurement. Therefore, the emphasis is on how to calibrate the distance *d* between the reference and the ground glass.

**Figure 3:**
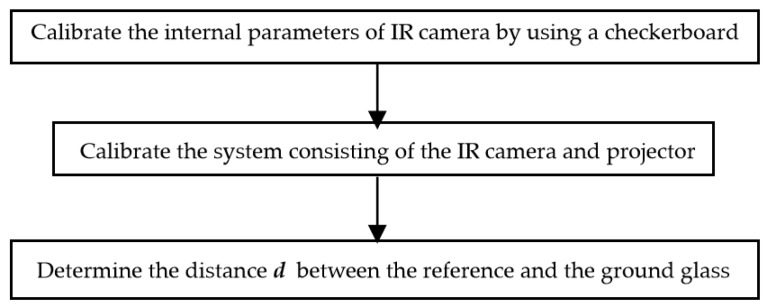
Flow chart of geometric calibration in IR-PMD.

The internal parameters of the IR camera were determined by a checkerboard, while the absolute phase and depth information of each pixel of the captured specular object were obtained by moving the sprayed plane mirror along the translating stage1. The procedure of calibrating the distance *d* involved the following steps, shown in Figure 3.

Step 1: Calibration of the internal parameters of the IR camera. The internal parameters include two focal lengths (F_u_ and F_v_), two principal point coordinates (P_u_ and P_v_), and four radial and tangential distortion coefficients (K_1_, K_2_, K_3_, and K_4_). The popular Camera Calibration Toolbox [29] was used to determine these parameters by a ceramic checkerboard. The checkerboard was positioned in several random positions and orientations in the measurement volume.

Step 2: Calibration of the relationship between the IR camera and the projector. Firstly, a white plate was moved to several known positions along the optical axis of the camera by another translating stage, called stage2, in the measurement volume. At each position, the projector projected IR sinusoidal fringe patterns to the white plate. Then, the IR camera captured the fringe patterns on the plate surface. Finally, the relationship between 3D shape and absolute phase could be determined by using a polynomial function [30]. Therefore, the polynomial coefficients of each pixel were calculated by the obtained depth data and absolute phase.

**Figure 4:**
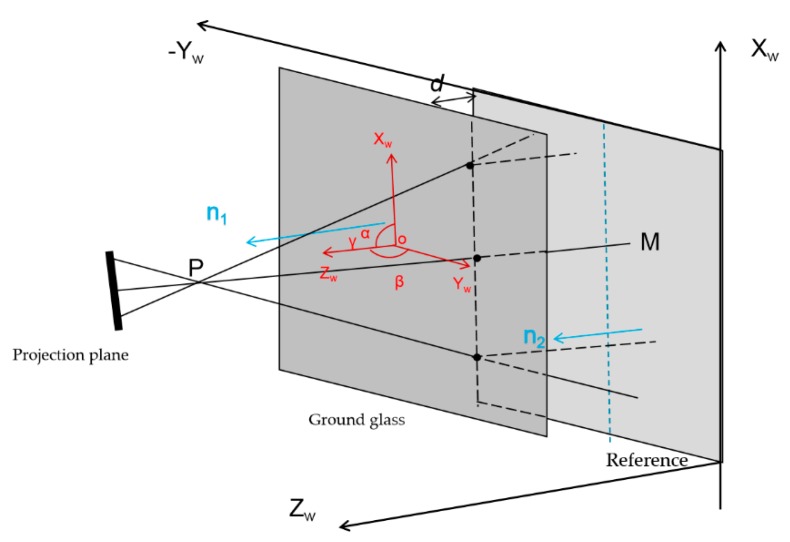
Schematic diagram of two-plane fitting.

Step 3: Calibration of the geometric relationship between the ground glass and the reference. Firstly, the sprayed mirror was placed in the position of the reference, and the ground glass was fixed in a different position; they were regarded as two measured objects. The IR camera captured the IR fringe patterns projected on both the ground glass and the mirror, as illustrated in Figure 2. Secondly, the ground glass and the mirror were adjusted so to be parallel. The sprayed mirror had to be adjusted perpendicular to the translating stage1. The least-square algorithm [31] was used to fit the plane of the ground glass and the mirror in the reference position. Plane equations for them are:

(6) $$a_{1}X+b_{1}Y+c_{1}Z+d_{1}=0$$

(7) $$a_{2}X+b_{2}Y+c_{2}Z+d_{2}=0$$

where a_i_, b_i_, c_i_, and d_i_ (i = 1,2) stand for the planar parameters, (X, Y, Z)^T^ denotes an arbitrary point on the plane in the camera coordinate system. It is easy and feasible to obtain the four planar parameters in Equations (6) and (7) with the least-square algorithm, then the normal vectors **n_1_** and **n_2_** on the two planes can be obtained, with **n_1_** = (a_1_,b_1_,c_1_), **n_2_** = (a_2_,b_2_,c_2_). The cosine angle of each plane’s normal vector **n** can be obtained by a reverse trigonometric function. A schematic diagram of the results of two-plane fitting is shown in Figure 4. PM represents the projecting axis of the projector, α, β, γ are the angles between a plane’s normal vector and the three coordinate axes X_W_Y_W_Z_W_, respectively. Assuming that the plane is perpendicular to the projecting axis of the projector, the normal vector is parallel to the projecting axis of the projector, i.e., OZ_W_//PM. Therefore, α = β = 90° and γ = 0°. As shown in Figure 4, on the basis of the angles α, β, γ of the reference, the orientations of the ground glass of the reference are adjusted to make them parallel to the each other. When adjusting the parallelism, it is necessary to calculate the normal vector by multiple-plane fitting and adjust it several times until the two planes are parallel. Thirdly, on the basis of the calibrated polynomial coefficients in Step 2, the coordinates XYZ of the ground glass and of the reference can be obtained. Finally, after the spatial positions of the reference and ground glass are adjusted, the two planes are fitted again, and the vertical distances from all points on the ground glass to the reference are calculated. An average value of all vertical distances is the relative depth *d*.

## 3. Experiments and Results

To test the proposed method, a hardware system was setup, and some experiments were carried out.

### 3.1. Hardware System

**Figure 5:**
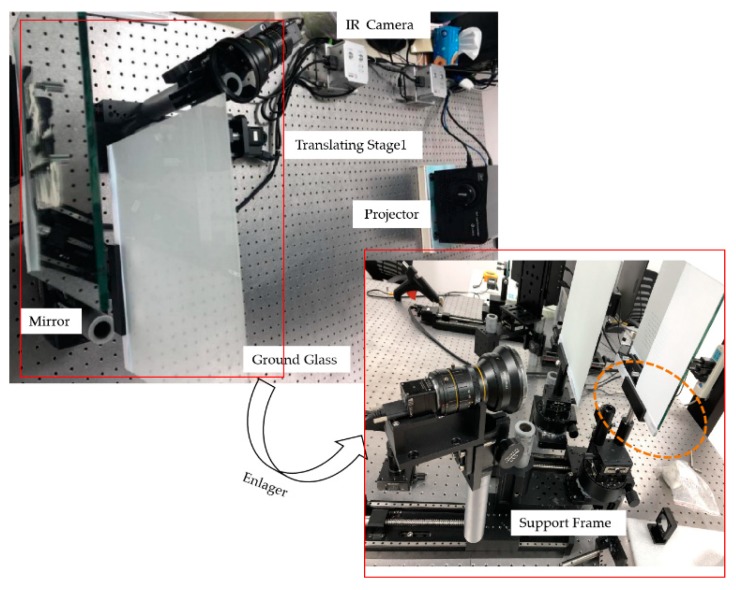
Hardware of the developed 3D measurement system.

In order to measure specular objects with isolated and/or discontinuous surfaces, a hardware system was developed, as illustrated in Figure 5. The system consisted of an IR camera (XIMEADE XIQ, MQ042RG-CM, with 2048 × 2048 pixels), a projector (TI, DLP Light Commander, with resolution 1024 × 768 pixels), a ground glass (Edmund Optics Worldwide, with size of 250 × 250 mm) with good diffusivity to light, high light transmittance, and a diffuse surface created by 220-grit sandblast, a translating stage1 (DHC, GCD-203300M, with the accuracy of 1 μm), and a support frame. The support frame was used to place the measured specular objects. The DLP Light Commander can project not only IR fringe patterns, but also visible fringe patterns. The translating stage1 moved the camera and the measured specular objects to two positions during the measurement. The camera used a standard prime lens with focal length of 12 mm and a filter on the front. The filter filtered out visible light and reduced the effects of ambient light when the IR camera captured IR fringe patterns. The projector used a manual focusing lens with a focal length of 50 mm. Internal parameters needed to be calibrated before the calibration of the system parameters.

The projector was placed in front of the ground glass. A mirror was manufactured by Jiaite Photoelectric Corporation, Shenzhen, China, for calibrating the parameter *d* of the IR-PMD system. The red line frame of Figure 5 shows a partial enlargement of the device, and the orange dashed line circle indicates the mirror whose surface was sprayed in part. The IR camera and the mirror were fixed together on the translating stage1, as illustrated in Figure 5. The IR camera should be fixed in a position on the translating stage1 that allows it to simultaneously see fringe patterns on both the ground glass and the sprayed mirror. The ground glass and the mirror were placed in parallel which should be adjusted during the experiment set up.

### 3.2. System Calibration

According to the previous geometric calibration procedure in sub-Section 2.2, the experimental system was calibrated by the following steps.

(1) Internal parameters of the IR camera.

A ceramic checkerboard with 168 squares (14 rows and 12 columns) having the same size of 10 mm × 10 mm and an accuracy of 0.005 mm, manufactured by Kailinbo Optical Technology Corporation, Shenzhen, China, was used to determine the internal parameters of the IR camera. The checkerboard was randomly placed 30 times in front of the IR camera, and the captured checkerboard images were used to calibrate the camera using the Camera Calibration Toolbox. The obtained internal parameters of the IR camera are shown in Table 1.

(2) Relationship between the IR camera and the projector.

A white plate was manufactured to calibrate the relationship between the IR camera and the projector. The plate was moved to 21 positions along another translating stage2 (also from DHC, GCD-203200M, with the accuracy of 1 μm). At each position, three sequence sinusoidal fringe patterns with numbers of 64, 63, and 56 [28], each sequence having four fringe patterns with π/2 phase-shifting between each other, were generated, sent to the projector, and then projected onto the plate surface. Then, the absolute phase data were obtained by using the optimum three-fringe number selection method. The depth information was obtained from the stage2. Therefore, the relationship between the IR camera and the projector was determined by the obtained phase data and the known distance moved.

(3) Parameters ∆*d* and *d* of the proposed IR-PMD system.

The parameter ∆*d* was determined by the translating stage1 during the procedure of measurement; *d* is the distance between the reference position and the ground glass. Through the proposed geometric calibration method in Section 2, *d* was determined by using the sprayed mirror.

### 3.3. Performance Analysis

After calibration of the geometric parameters, some experiments were carried out by measuring an artificial mirror step to evaluate the performance of the proposed method.

**Figure 6:**
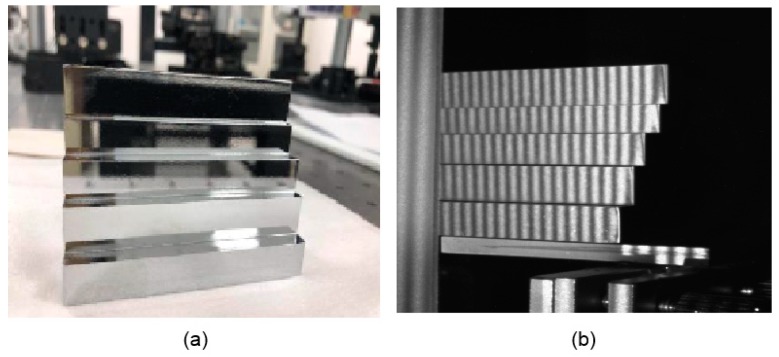
Captured patterns of an artificial mirror step. (**a**) Photo of the mirror step; (**b**) Captured infrared patterns reflected by the mirror step.

**Figure 7:**
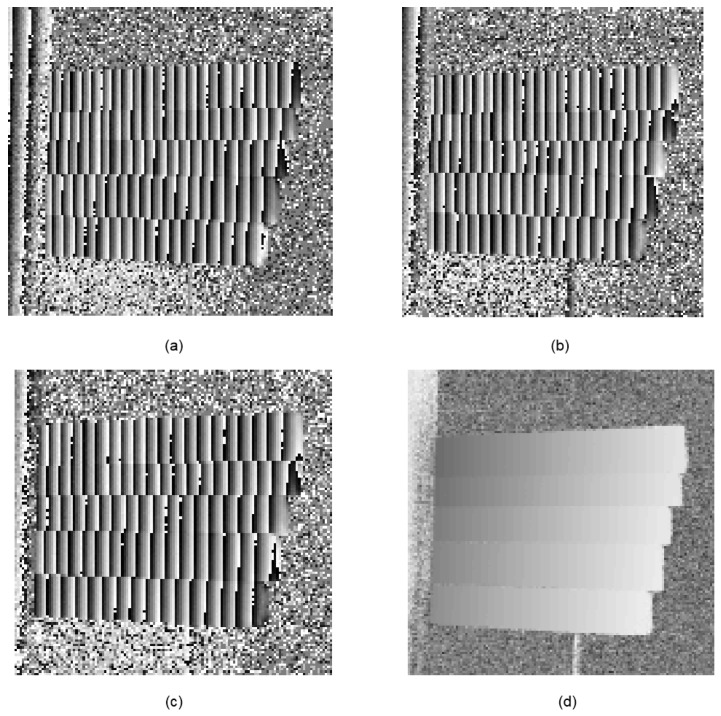
Phase maps of the artificial mirror step. (**a**)–(**c**) Wrapped phase of the step; (**d**) Unwrapped phase of the step.

**Figure 8:**
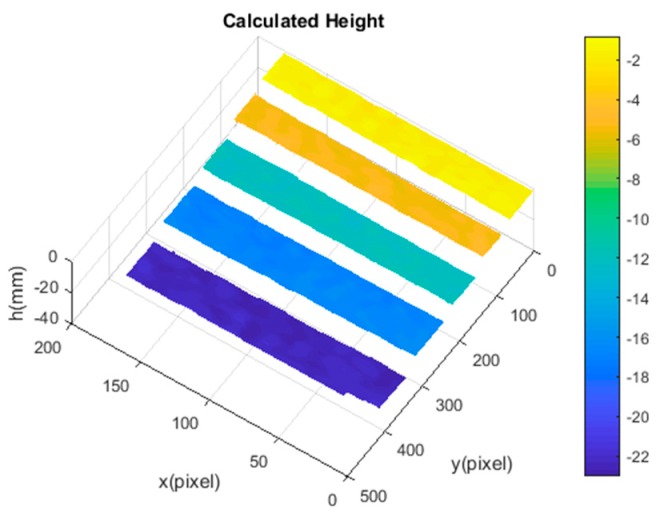
3D shape data of the artificial mirror step.

An artificial mirror step having discontinuous specular surfaces was manufactured, as illustrated in Figure 6a. The distance between neighbor step surfaces of the artificial mirror step was measured by a coordinate-measuring machine (CMM, with a measuring error of 2 μm) beforehand, as shown in Table 2, second column. Three sequence sinusoidal fringe patterns with numbers of 64, 63, and 56 [28], each sequence having four fringe patterns with π/2 phase-shifting between each other, were generated, sent to the projector, and then projected onto the ground glass. The IR fringe patterns were reflected by the measured specular surfaces, and the deformed fringe patterns were captured by the IR camera, as illustrated in Figure 6b. The four-step phase-shifting algorithm and the optimum three-fringe number selection method were applied to the deformed fringe patterns to obtain the wrapped and unwrapped phase data, as illustrated in Figure 7a–d, respectively. In order to reduce the phase error, we carried out the following steps: the camera’s gamma value was set to 1 to eliminate the camera’s non-linear effects, and the lens distortion of the camera was eliminated while calculating the phase data to reduce the influence of measurement errors; a filter was used on the front of the camera to reduce the influence of ambient light; the phase data were filtered to reduce the random noise of phase error without affecting the phase information. Using the calibrated parameters, 3D shape data of the artificial mirror step were obtained, as illustrated in Figure 8.

The least-square algorithm was used to fit all measured points of a step surface into a plane. The measured distances between the neighbor steps were calculated as the neighboring fitted planes, as shown in the third column of Table 2. The absolute errors were below 21.3 μm, as listed in the fourth column of Table 2. The experimental results validated the effectiveness and accuracy of the proposed method.

### 3.4. Experimental Results

#### 3.4.1. Measurement Results

**Figure 9:**
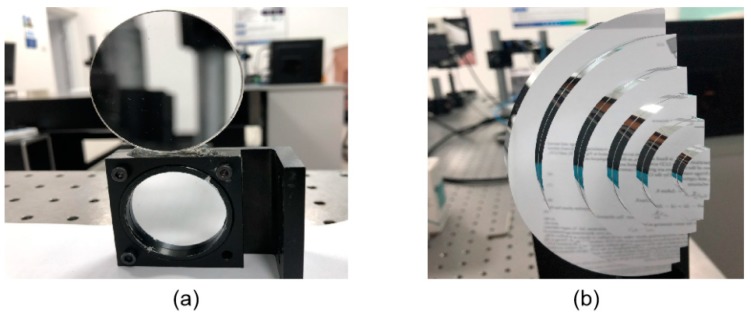
Photos of the measured specular objects. (**a**) Two separate specular objects; (**b**) Discontinuous fan-shaped mirror step.

**Figure 10:**
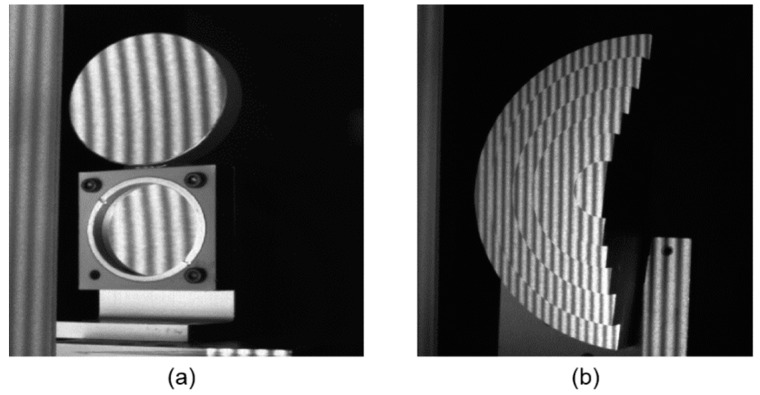
Captured fringe patterns of the measured specular objects. (**a**) Two separate specular objects; (**b**) Discontinuous fan-shaped mirror step.

**Figure 11:**
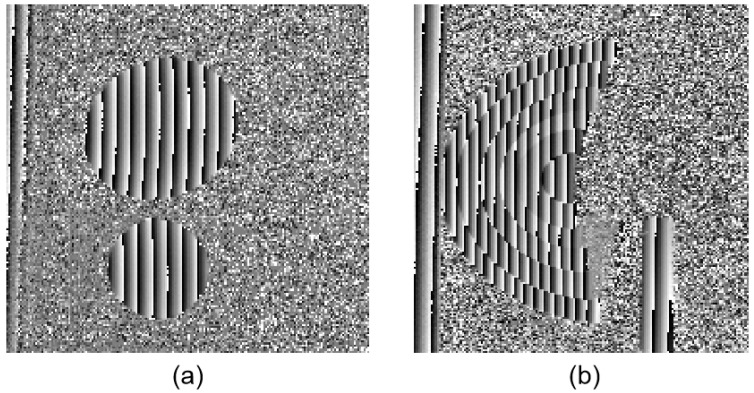
Wrapped phase maps of the measured specular objects. (**a**) Two separate specular objects; (**b**) Discontinuous fan-shaped mirror step.

**Figure 12:**
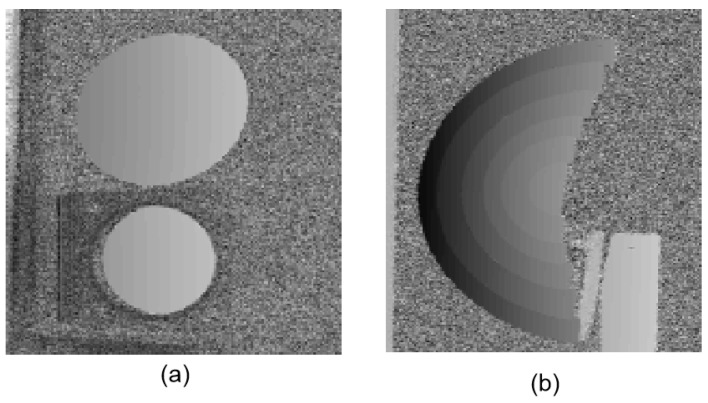
Unwrapped phase maps of the measured specular objects. (**a**) Two separate specular objects; (**b**) Discontinuous fan-shaped mirror step.

Two separated freeform mirrors and a discontinuous fan-shaped mirror step were measured by the calibrated system, as illustrated in Figure 9a,b. The fan-shaped mirror step was manufactured by Shanghai Engineering Research Center of Ultra-Precision Optical Manufacturing in Fudan University, and the distance between the neighbor step surfaces of the fan-shaped mirror was known beforehand, as shown in the second column of Table 3. Three sequence sinusoidal fringe patterns with numbers of 64, 63, and 56 [28], each sequence having four fringe patterns with π/2 phase-shifting between each other, were generated by software and projected onto the ground glass by the projector. The IR fringe patterns on the ground glass were reflected by the measured specular surfaces. The reflected deformed IR fringe patterns were captured by the IR camera from another viewpoint, as illustrated in Figure 10a,b. Three wrapped phase maps were calculated by using the four-step phase-shifting algorithm, as shown in Figure 11a,b. Figure 12a,b shows the absolute phase maps by using the optimum three-fringe number selection method.

**Figure 13:**
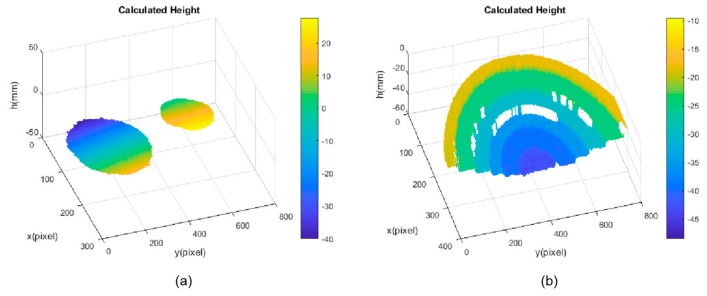
3D shape data of the measured specular objects. (**a**) Two separate specular objects; (**b**) Discontinuous fan-shaped mirror step.

By using the calibrated parameters, the 3D shape data of the measured specular objects were obtained, as illustrated in Figure 13a,b. The results demonstrate that the proposed method can directly measure specular objects having isolated and/or discontinuous surfaces.

#### 3.4.2. Comparison Analysis of IR and Visible Fringe Patterns

In order to further quantitatively evaluate the performance of the proposed IR-PMD method, visible fringe patterns were projected to measure the fan-shaped mirror step, as illustrated in Figure 9b. The four-step phase-shifting algorithm and the optimum three-fringe number selection method were applied to obtain the wrapped and unwrapped phase data from the captured IR and visible fringe patterns. The 3D shape of the fan-shaped mirror step was obtained by using the calibrated geometric parameters. The distance between the neighbor steps was calculated as the neighbor fitted planes. The step height, the actual distances between the neighbor steps of the fan-shaped mirror step, are listed in the first and second columns of Table 3. The third and fourth columns of Table 3 are the measured distance between neighbor step surfaces determined by using IR light and visible light, respectively. The results of the measured step depth data were compared with the actual heights to obtain the absolute error. Table 3 shows the absolute errors of the measurement results by IR light and visible light in the fifth and sixth column, respectively. For any two neighbor steps, the absolute errors measured by IR light were much smaller than those obtained by measuring with visible light, as the fifth and sixth column illustrate. It can be see that the reconstructed 3D shape of the specular surfaces was sensitive to ambient light and was affected by the external environment. Therefore, the proposed IR-PMD method can reduce the influence of ambient light and improve the measurement accuracy.

## 4. Conclusions

This paper presents a new full-field 3D shape measurement method by using IR-PMD to obtain specular components having discontinuous surfaces. The fringe projection and fringe reflection technology were used to build the direct relationship between the absolute phase data and the depth data, without the procedure of gradient integration. The proposed system applies an IR light source to generate IR sinusoidal fringe patterns, so that the effects of ambient light can be reduced, and enough reflection on many specular surfaces can be created. A projector projecting IR sinusoidal fringe patterns onto a ground glass is regarded as an IR digital screen. An IR camera with the measured specular surfaces was moved simultaneously to two positions by a translating stage to realize two screen designs. In addition, a new calibration method was proposed to build the direct relationship between the phase map and the depth data. A mirror was used for system parameters calibration. Part of the mirror surface was sprayed for the purpose of using the fringe projection technique to determine its orientation in the camera coordinate system. After the absolute phase information was obtained, the depth data of the measured specular objects could be directly calculated by a derived equation without gradient integration. The effectiveness and accuracy of the proposed method were validated by some experiments on an artificial mirror step and two discontinuous specular objects.

The proposed method has the following two advantages. On one hand, IR light is used as a light source to enhance the measurement accuracy because it is insensitive to the effect of ambient light. On the other hand, the proposed IR-PMD method was designed to develop a direct relationship between phase map and depth data by establishing a new mathematical model, so that it can measure specular objects having discontinuous and/or isolated surfaces and avoid the procedure of gradient integration.

In the near future, we will study the effects of error sources, including nonlinear influence, lens distortion of imaging and projecting system, geometric calibration error, on the measurement results and evaluate the performance of the measurement system.

## Figures and Tables

**Table 1:** Internal parameters of the camera.

Name	Calibration Results
Focal length	fc = [2355.03482, 2355.59838] ± [1.47732, 1.46593]
Principal point location	cc = [1016.92767, 1018.56904] ± [0.27911, 0.25847]
Lens distortion coefficient	kc = [−0.13744, 0.14780, −0.00004, −0.00016, 0.00000] ± [0.00044, 0.00308, 0.00002, 0.00002, 0.00000]
Pixel error	err = [0.03298, 0.02954]

**Table 2:** Experimental results of the mirror step (units: mm).

Step Height	Actual Distance	Measured Distance	Absolute Error
1–2	3.9868	4.0081	0.0213
2–3	7.0248	7.0213	0.0035
3–4	5.0062	5.0141	0.0079
4–5	6.0986	6.1180	0.0194

**Table 3:** Comparison results of the fan-shaped mirror step by using IR light and visible light (units: mm).

Step Height	Actual Distance	Measured Distance		Absolute Error	
		IR Light	Visible Light	IR Light	Visible Light
1–2	6.50038	6.50312	6.52437	0.00574	0.02399
2–3	5.50026	5.49593	5.47372	0.00447	0.02654
3–4	500026	4.98955	4.96414	0.01071	0.03612
4–5	4.00015	3.99248	4.02947	0.00767	0.02932
5–6	3.00016	3.01798	3.03436	0.01782	0.03420
